# “Percorso Giacomo”: An Italian Innovative Service of Perinatal Palliative Care

**DOI:** 10.3389/fped.2020.589559

**Published:** 2020-11-19

**Authors:** Chiara Locatelli, Luigi Corvaglia, Giuliana Simonazzi, Maria Bisulli, Lucia Paolini, Giacomo Faldella

**Affiliations:** ^1^Neonatal Intensive Care Unit, S.Orsola-Malpighi University Hospital, Bologna, Italy; ^2^Department of Medical and Surgical Sciences (DIMEC), University of Bologna, Bologna, Italy; ^3^Obstetric Unit, Department of Medical and Surgical Sciences, Policlinico Sant'Orsola-Malpighi, University of Bologna, Bologna, Italy; ^4^University of Bologna, Bologna, Italy

**Keywords:** perinatal palliative care, neonatal palliative care, life-limiting conditions, comfort care, interdisciplinary team

## Abstract

The perspective proposed by this article will focus on perinatal palliative care as a strategy for improving the quality of life of neonates with life-limiting conditions when extending the patient's life is no longer the goal of care. This manuscript reports the creation of an innovative program of perinatal palliative care called “Percorso Giacomo” (Giacomo's Pathway) at Sant'Orsola Hospital in Bologna, Italy in 2013. Key features include interdisciplinary collaboration between professionals from obstetrics, neonatology and other specialties aiming to reach the most detailed fetal and neonatal diagnosis and prognosis; communication and engagement with the family to discuss goals of care and prepare a birthing plan that follows the family's desires and expectations; and personalized care to achieve comfort for each newborn and support for families according to their social, cultural, and religious backgrounds.

## Introduction

Perinatal palliative care (PPC) is offered in the settings of fetal or neonatal diagnoses of life-limiting conditions (LLCs) as a plan to achieve comfort of the neonate and to support the family. Given the complexity of the medical and non-medical needs of these neonates and the emotional challenges for parents, PPC is a necessarily holistic and interdisciplinary approach to care. PPC is a journey with families that begins at the moment of diagnosis, whether prenatal or postnatal, and involves a collaboration among maternal-fetal medicine specialists, neonatologists, and other professionals with the goal of establishing an individualized plan of care for the mother-baby dyad.

The American College of Obstetricians and Gynecologists (ACOG) considers PPC one of the options of care that should be offered to patients facing pregnancies complicated by fetal LLC (1). Yet, PPC is not yet routinely integrated in prenatal counseling in the United States (2, 3). This also holds true for Italy. A recent Italian national survey of neonatal intensive care unit (NICU) practitioners demonstrated that only 30% of the institutions represented offer a structured PPC program. Of these, 34% have only one program coordinator, and fewer than 30% organize PPC training for health care providers (4).

Despite technological advances in the ability to detect severe fetal anomalies, infants with LLCs are occasionally diagnosed only after birth. Moreover, critically ill neonates admitted to the NICU can face a potentially adverse prognosis or reach the end-of-life stage, at which point a redirection of goals of care is required. According to the World Health Organization (WHO), palliative care and intensive care should be provided concurrently to critically ill infants with potentially adverse prognoses (5). The American Academy of Pediatrics (AAP) and the Italian Society of Neonatology (SIN) provide recommendations for achieving comfort of terminally ill neonates and of those affected by an LLC (6–9).

The urgent need to provide the option of PPC to families with neonates prenatally or postnatally diagnosed with an LLC prompted the creation and implementation of an innovative PPC program at Sant'Orsola Hospital (SOH) in Bologna in 2013. The program was named “Percorso Giacomo” (Giacomo's Pathway) in honor of the first infant to whom SOH provided such care. This paper describes the origin and the development of the program over its first 7 years.

## Clinical Case

Giacomo was prenatally diagnosed with anencephaly. His parents, although aware of his short life expectancy, chose to carry the pregnancy to term. The mother was a G4 P3 woman with two healthy children and another baby with anencephaly whose delivery was complicated by failure to progress and stillbirth. As she again received an anencephaly diagnosis, she asked her provider to manage her care with the goal of having some precious time with her baby after birth. At that time there were no guidelines for PPC at SOH, so the obstetrician involved a neonatologist and a midwife to plan the delivery and the infant's postnatal care according to the mother's desire. Giacomo was delivered by cesarean section (transverse position) with Apgar scores of 9 and 9. After his scalp wound was carefully dressed, he was given to his family to hold. Per parental wish, he was baptized by the hospital chaplain. Following the family desire for bonding, Giacomo was allowed to room-in with his mother in a private postpartum room. At SOH, this was the first time that a baby with LLC was not admitted to the NICU. Comfort measures, including skin-to-skin care and holding to facilitate bonding and warmth, were offered. The infant had no sucking reflex; thus, he was given small feeds via an orogastric tube to relieve hunger and thirst. Discomfort and pain were assessed but Giacomo had no signs of distress and did not need any pharmacological treatment. Giacomo lived for only 19 hours, but he had the opportunity to meet his parents, siblings, and relatives, and he was comfortable throughout his entire life.

Written informed consent was obtained from Giacomo's parents for the publication of any potentially identifiable images or data included in this article.

## Percorso Giacomo

### Origin

The experience with Giacomo made our perinatal team aware of the urgency to formalize a systematic approach to families who received a fetal or neonatal diagnosis of LLC, and a collaborative journey began. Another key factor that triggered the initiation of a PPC program, was that the neonatologist who provided care to Giacomo received training in PPC in 2009–2011 by participating in the Neonatal Comfort Care Program (NCCP) (10) at Columbia University Medical Center (CUMC) in New York, NY, US. Upon her return to Italy she began to investigate the feasibility of a similar program at SOH, and the meeting with Giacomo's family was the occasion to start the project.

During the debreafing of Giacomo's case, each of the professionals involved in the care expressed interest in developing a service for similar cases and the planning began. The group decided to name the program “Percorso Giacomo” (PG) in honor of Baby Giacomo.

The experience with this family was not only the trigger for the planning of a new service, but also a milestone in the understanding of the complex medical and non-medical needs of these infants and their families.

The group then opened to other professionals interested in the same goal, and a working group of champions called the “Percorso Giacomo Team” (PGT) was established. Two main objectives were identified: (1) assemble an interdisciplinary team, and (2) define guidelines and eventually establish a new hospital policy for PPC.

### Gathering an Interdisciplinary Team

SOH is a large academic institution with a busy maternal-fetal medicine service that has approximately 3,500 deliveries each year. The level 3 NICU takes care of some 300 critically ill neonates annually, including infants prenatally or postnatally diagnosed with an LLC. The two services work collaboratively, but until 2013 there was limited knowledge and no direct experience of PPC.

The PGT is an interdisciplinary team that includes nine clinicians from obstetrics and neonatology who identify themselves as champions for the care of mothers and infants with a fetal or neonatal diagnosis of LLC. The program is not funded, and the team members make themselves available as primary providers for the mother-baby dyad and facilitate the execution of the plan of care in collaboration with medical staff involved in the care. Professionals from a variety of other specialties (cardiology, neurology, surgey, nephrology, genetics, etc.) are also available for consultation.

#### Establishing Guidelines

After carefully reviewing PPC literature, including recommendation from the ACOG (11), the AAP (6), and the SIN (8), and through networking with other national and international academic centers, local guidelines were created and implemented to standardize interventions. The essential elements are summarized in Tables 1, 2.

**Table 1:** Guidelines to provide comfort to neonates prenatally diagnosed with life-limiting conditions.

**At prenatal counseling**
• Discuss comfort measures in detail and assure parents that a state of comfort for their baby can always be achieved, regardless of his/her condition • Fill out a birthplan with the family according to their preferences and cultural/social/religious background and give a copy to family • Obtain pictures/videos of fetal ultrasounds as per family's wishes • Discuss administration of Vitamin K, eye prophylaxis
**Before delivery**
• Review birthplan with family • Collect items for special dressing if needed (anencephaly, limb-body-wall complex, etc.) and clothing appropriate for the baby's clinical condition and size • In case a blood test is needed to confirm the diagnosis, plan for cord sample
**After delivery**
• Evaluation of baby's clinical status, provide gentle suction if needed • Place hat, place dressing as needed and wrap in clean warm blanket • Allow mother and/or father for skin-to-skin or holding • Facilitate bonding with siblings and extended family as per family's wishes • Involve chaplain as per family's wishes • Obtain pictures, videos and memories as per family's wishes • Transfer mother and baby to post-pastum for rooming-in in a private room
**Neonatal plan of care**
• Vital signs: heart rate, respiratory rate, temperature and pain score (NIPS or EDIN) every 3 h or as needed according to the baby's status • No blood or other tests should be obptained, unless needed for confirmation diagnosis • Encourage breastfeeding or gavage feeds as appropriate. Establish a minimum intake to guarantee relief of hunger/thirst (breast milk or formula). Consider colostrum care for babies at end of life • Assessment of respiratory distress (air hunger, agitation, increased work of breathing, gasping) and use of non-pharmacological strategies (gentle suctioning upper airways, positioning) or pharmacological treatment (morphine sulfate PO, lorazepam PO)

*NIPS, Neonatal Infant Pain Scale; EDIN, Echelle Douleur Inconfort Nouveau-né; PO, per os*.

**Table 2:** Guidelines to provide comfort to neonates with life-threatening or terminal conditions in the NICU.

**Neonates critically ill but still receiving full intensive care**
• Discuss baby's clinical status and potential comfort interventions with medical/nursing team • Discuss with parents a plan of comfort interventions to improve quality of life • Provide privacy in the NICU • Facilitate baby/parents bonding by holding, gentle touch, massage • Encourage breast pumping if mother desires to give her baby breast milk • Provide parents the opportunity to take care of their baby's needs (colostrum care, non nutritive sucking, diaper change, sponge bath, dressing, etc.) • Involve siblings and extended family as per family's wishes • Involved chaplain as per family's wishes • Obtain pictures, videos and memories as per family's wishes
**Neonates at end-of-life stage after redirection from intensive to palliative care**
• Discuss plan for weaning of life-sustaining support with parents • Assure parents that a state of comfort for their baby can always be achieved, regardless of his/her condition • Encourage parents to stay/hold the baby • Involve chaplain as per family's wishes • Involve siblings and extended family as per family's wishes • Obtain pictures, videos and memories as per family's wishes • Provide private settings with intermittent supervision of medical/nursing team • Wean life-sustaining support, silence monitors'alarm, remove unnecessary devices and IV/arterial lines in appropriate fashion • Maintain an IV access, if present, for pain management • Facilitate bonding and warmth by skin-to-skin, holding, colostrum care • Assess signs of respiratory distress (air hunger, agitation, increased work of breathing, gasping) and assess pain by validated clinical scores (NIPS or EDIN) • Use non-pharmacological strategies (gentle suctioning upper airways, positioning) or pharmacological treatment (acetaminophen PO; morphine sulfate PO/IV; fentanyl IV; lorazepam PO/IV; midazolam PO/IV) • After the baby dies, help parents to sponge bath and dress their baby • Discuss with family in regard to autopsy consent

*IV, intravenous; NIPS, Neonatal Infant Pain Scale; EDIN, Echelle Douleur Inconfort Nouveau-né; PO, per os*.

As the focus of care is the satisfaction of neonates' basic needs for bonding, maintanance of body temperature, and relief of hunger and thirst, our guidelines reflected these recommendations. Pain and distress are assessed and treated with pharmacological interventions (12). The Neonatal Infant Pain Scale (NIPS) (13) and Echelle Douleur Inconfort Nouveau-ne' (EDIN) (14) are the preferred pain assessment tools because they are based on the infant's behavior and do not require vital signs detection, which could disrupt the bonding experience. Each neonate has unique medical and non-medical needs; thus, we personalized the care plan in consideration of diagnosis, prognosis, gestational age, and family preferences. Additionally, as a result of a policy change in our hospital, when a neonate, prenatally diagnosed with an LLC, is not admitted to the NICU, is directly transitioned from delivery room to a post-partum for rooming-in with the mother in private setting. The midwife in charge of the mother-baby dyad provides support and care in collaboration with NICU nurses and neonatologist is available for medical evaluation and interventions.

#### Criteria for Enrollment

PG offers PPC to neonates prenatally or postnatally diagnosed with an LLC that is expected to render their life brief, such as renal agenesis, anencephaly or extreme prematurity. PPC is also offered to neonates diagnosed with severe genetic, metabolic, skeletal, cardiac, neurological or oncological conditions and life-limiting syndromes. The provision of intensive care can prolong the life of these neonates, but when the burden of invasive and aggressive treatment might exceed the benefit in terms of length of life, an option of PPC is offered.

#### Perinatal Counseling

The PGT can be accessed in different ways. If a mother receives a diagnosis of fetal LLC at SOH, the obstetric provider offers available options, including consultation with the PGT. Patients might also be referred by outside institutions. After consultation, the mother has the option to transfer her care to SOH, or to continue care with her current provider, with the PGT functioning as consultant.

Members of the PGT—including a neonatologist, an obstetrician, and a midwife—are involved in the encounter with the family. Before meeting the family, team members meet to review available information about the fetal diagnosis, discuss the need to obtain further tests and/or to involve more specialists, and identify care options to be presented to the family. It is essential that the family receives clear and honest information from both obstetricians and neonatologists.

The conversation with the family focuses on communicating of detailed information about the presumed or certain diagnosis and prognosis and, if needed, any further medical investigations. Team members listen attentively to the family to identify concerns, hopes, and expectations that will help them prepare a comprehensive plan for the reminder of the pregnancy, labor and delivery, and postnatal birthing plan. At the end of the meeting the parents are also given the option to meet a psychologist to assess mental health and to provide grief support, resources, and referrals for the family at large. Depending on the complexity of the diagnosis and the gestational age, one or more meetings are needed to complete a comprehensive plan.

The outcome of the conversation/s is documented on the mother's health record to facilitate communication with all clinicians involved in mother-baby care. Moreover, providers and family complete a document -the “birthing plain”- that is kept by the family and presented upon hospital admission.

Members of the PGT follow labor and delivery, and the neonatologist present at birth either confirms the neonate's diagnosis or obtains further tests for confirmation. The team ensures that the birth plan formulated during prenatal counseling is included in the overall management care plan.

When neonates admitted to the NICU are postnatally diagnosed with an LLC or a potentially adverse prognosis, the medical team attentively evaluates each case and may involve the PGT as an extra layer of support to families. Different options can be proposed including the continuation of intensive care with focus on quality of life, or the redirection of goals of care with transition to palliative care.

#### Pregnancy and Neonatal Outcomes

The PGT cared for 24 cases in its first 7 years. The team followed 20 pregnancies, the outcome of which included two intrauterine fetal deaths, two second trimester spontaneous abortions, and 16 live births. Three of these infants are currently alive and stable, and PPC was not considered medically appropriate for these infants because their postnatal diagnoses were more favorable than had been expected. The PGT also facilitated palliative care plans for four neonates admitted to the NICU with severe brain injury secondary to severe metabolic conditions or hypoxic-ischemic insults, all of whom died.

#### Education and Program Development

Teaching and training are essential to fostering a mindset that includes palliative care as an essential component of medical practice in perinatology. Over time the PGT built several initiatives for PPC training at SOH. Formal teaching, including a curriculum of six lessons for pediatric residents and fellows, was established in 2017. Bedside training and clinical case discussion for physicians, midwives, and nurses also have been introduced over the past 7 years.

In 2014, the concern for education and program development inspired the PGT to organize a National Workshop featuring national and international experts in PPC. The workshop goals included presentation of cutting-edge experiences of PPC and network development. The workshop was attended by some 300 clinicians from all across Italy and laid the foundation for collaboration with several academic institutions. The meeting was also attended by the Maternal Infant Department leadership and administrators. They expressed their interest and encouraged development of a PPC service at SOH. The PGT then worked tirelessly with administrators and leadership to establish a new hospital policy for PPC, which was approved in 2019.

In our region of Italy, Emilia-Romagna, a new project for the organization of a regional palliative care network has begun (15). This pediatric hospice network offers inpatient care and home care for children with complex medical conditions and chronic health problems. PG has been included in this network by the project committee. This is an essential step to build a continuity of care for neonates born with LLC or for those with complex diseases and potentially adverse prognoses. After hospital discharge, infants can be supported either at home or in a rehabilitation facility by specialists in palliative care. This project is also an opportunity to promote the palliative care mindset in our region and, hopefully, in the entire nation.

## Discussion

Over the past years, PPC has been gaining interest in Italy, and clinicians involved in perinatology have been working to define the best care practice (16) and to establish adequate pathways to care for neonates diagnosed with LLCs (17). Moreover, the SIN has offered suggestions for PPC in a document focused on guidelines for prevention and treatment of neonatal pain (8, 9) and Italy has enacted legislation promoting the use of palliative care (18).

PG has positioned itself as an innovative pathway for PPC origins and guidelines.

What triggered the actualization of this program was the desire of a family (Giacomo's parents) to be accompanied along the perinatal journey. The professionals involved in the care were inspired to create a standardized program for families with similar needs. The experience of PG show that a strong motivation is pivotal to the birth of a new program.

PG was modeled after the NCCP in New York. In the planning process the PGT utilized evidence-based recommendations (6, 8, 9, 11); however, the innovative aspects of the PG guidelines came largely from direct experience and ongoing collaboration with the NCCP. An important step for “culture change” at SOH is the transition of neonates with LLC from delivery room directly to post-partum to room-in with the mother, instead of admission to the NICU (Table 1).

Another innovative element includes the introduction of early palliative care interventions to improve quality of life of babies still receiving full intensive care (Table 2). Both innovations have been supported by studies of parental satisfaction (19) and stress evaluation (20) published by the NCCP.

In pregnancy care, two patients are at stake. Thus, tight collaboration of specialists in obstetrics and neonatology is essential to propose available options (1), to plan a safe pregnancy and delivery, and to provide a personalized care for each neonate in accord with the family's preference (21). Pregnancy is a time of hope and expectation, but with the discovery of severe anomalies, fear can quickly overcome hope as expectations are no longer the norm. The option of PPC supports the family and helps in the decision-making process (22, 23). Moreover, the 7-years experience of PG shows that families felt welcomed and embraced along the perinatal journey. Parents expressed of PG gratitude and praise for the program, as clearly illustrated by parental feed-back messages. “*PG was the light at the end of the tunnel, PG was the hope that our baby would have been able to be embraced by mom, dad an her siblings”*; “*PG taught us that our life cannot be measured by its lenght, rather by the mark we leave”*; “*The program filled somehow the void we experienced and we felt serene.”*

The greatest desire of parents of neonates with LLC is not wanting them to suffer. Thus, pain and distress must be attentively assessed and treated (12). In the population we served any degree of discomfort was successfully managed with the use of non-pharmacological strategies, such as skin-to-skin holding, and colostrum care (24) or proper medications. This observation has been validated by studies of parental perception of their infants' state of comfort, when infants were treated with standardized comfort measures (19).

PPC not only addresses the population of neonates expected to have early perinatal demise, but also can be integrated with the intensive care of critically ill neonates at any stage of the illness (20, 25). In its first years, PG focuses largely on the care of mothers with fetal life-limiting diagnoses and followed neonates with short life expectancy however, in time the PGT began to be consulted regarding the plan of care of critically ill neonates with unexpected life-threatening diagnoses. Thus the PGT developed guidelines to facilitate quality of life during the NICU admission and/or to redirect goals of care at end of life (Table 2).

## Future Direction

PG has been offering perinatologists the opportunity to improve their confidence in this growing field in medicine. Within 1 year of the program's establishment, the PGT organized a national conference. Since that time many other educational initiatives have been proposed to guide and educate providers. When PPC knowledge is lacking, clinicians are uncomfortable and reluctant to offer this much-needed care (26). Thus, PG is planning to continue to offer training courses for medical and nursing professionals built on the knowledge and experience acquired over the past 7 years.

Throughout the years, PG has followed infants with a variety of severe life-threatening conditions, including rare and complex diseases. Collaboration with other academic centers has been essential to share experiences, to discuss challenging cases, and to provide the best possible care to each infant. Further networking opportunities will support the implementation of new programs in tertiary NICUs and will provide the necessary tools to professionals working in small institutions where programs cannot be implemented.

PG intends to facilitate the spreading of a mindset that integrates palliative care in the medical plan for neonates with LLC or critically ill. Being aware that further studies measuring outcomes and quality of care are needed, our group is in the process of analyzing the efficacy of this program.

As recommended by national and international organizations (1, 6, 27), PPC is a crucial option to be offered to families facing a fetal or neonatal diagnosis of LLC. Essential components include early involvement with families at the time of diagnosis, interdisciplinary collaboration of perinatologists, and promotion of education to facilitate a culture change.

## Data Availability Statement

The original contributions presented in the study are included in the article/supplementary material, further inquiries can be directed to the corresponding author/s.

## Ethics Statement

Written informed consent was obtained from the participant's parents for the publication of any potentially identifiable images or data included in this article.

## Author Contributions

CL conceptualized and designed the report. MB, LC, GF, LP, and GS drafted the initial manuscript and approved the final manuscript as submitted. All authors contributed to the article and approved the submitted version.

## Conflict of Interest

The authors declare that the research was conducted in the absence of any commercial or financial relationships that could be construed as a potential conflict of interest.
